# Combination of Radiological and Clinical Baseline Data for Outcome Prediction of Patients With an Acute Ischemic Stroke

**DOI:** 10.3389/fneur.2022.809343

**Published:** 2022-04-01

**Authors:** Lucas A. Ramos, Hendrikus van Os, Adam Hilbert, Silvia D. Olabarriaga, Aad van der Lugt, Yvo B. W. E. M. Roos, Wim H. van Zwam, Marianne A. A. van Walderveen, Marielle Ernst, Aeiko H. Zwinderman, Gustav J. Strijkers, Charles B. L. M. Majoie, Marieke J. H. Wermer, Henk A. Marquering

**Affiliations:** ^1^Department of Biomedical Engineering and Physics, Amsterdam University Medical Centers, University of Amsterdam, Amsterdam, Netherlands; ^2^Department of Clinical Epidemiology and Data Science, Amsterdam University Medical Centers, University of Amsterdam, Amsterdam, Netherlands; ^3^Department of Neurology, Leiden University Medical Center, Leiden, Netherlands; ^4^CLAIM - Charité Lab for Artificial Intelligence in Medicine, Charité Universitätsmedizin Berlin, Berlin, Germany; ^5^Department of Radiology and Nuclear Medicine, Erasmus Medical Center (MC) - University Medical Center, Rotterdam, Netherlands; ^6^Department of Neurology, Amsterdam University Medical Centers, University of Amsterdam, Amsterdam, Netherlands; ^7^Department of Radiology, Cardiovascular Research Institute Maastricht, Maastricht University Medical Center, Maastricht, Netherlands; ^8^Department of Radiology, Leiden University Medical Center, Leiden, Netherlands; ^9^Centre for Radiology and Endoscopy, Department of Diagnostic and Interventional Neuroradiology, University Medical Centre Hamburg-Eppendorf, Hamburg, Germany; ^10^Department of Radiology and Nuclear Medicine, Amsterdam University Medical Centers, University of Amsterdam, Amsterdam, Netherlands

**Keywords:** ischemia stroke, radiomics, deep learning, data combination, outcome prediction

## Abstract

**Background:**

Accurate prediction of clinical outcome is of utmost importance for choices regarding the endovascular treatment (EVT) of acute stroke. Recent studies on the prediction modeling for stroke focused mostly on clinical characteristics and radiological scores available at baseline. Radiological images are composed of millions of voxels, and a lot of information can be lost when representing this information by a single value. Therefore, in this study we aimed at developing prediction models that take into account the whole imaging data combined with clinical data available at baseline.

**Methods:**

We included 3,279 patients from the MR CLEAN Registry; a prospective, observational, multicenter registry of patients with ischemic stroke treated with EVT. We developed two approaches to combine the imaging data with the clinical data. The first approach was based on radiomics features, extracted from 70 atlas regions combined with the clinical data to train machine learning models. For the second approach, we trained 3D deep learning models using the whole images and the clinical data. Models trained with the clinical data only were compared with models trained with the combination of clinical and image data. Finally, we explored feature importance plots for the best models and identified many known variables and image features/brain regions that were relevant in the model decision process.

**Results:**

From 3,279 patients included, 1,241 (37%) patients had a good functional outcome [modified Rankin Scale (mRS) ≤ 2] and 1,954 (60%) patients had good reperfusion [modified Thrombolysis in Cerebral Infarction (eTICI) ≥ 2b]. There was no significant improvement by combining the image data to the clinical data for mRS prediction [mean area under the receiver operating characteristic (ROC) curve (AUC) of 0.81 vs. 0.80] above using the clinical data only, regardless of the approach used. Regarding predicting reperfusion, there was a significant improvement when image and clinical features were combined (mean AUC of 0.54 vs. 0.61), with the highest AUC obtained by the deep learning approach.

**Conclusions:**

The combination of radiomics and deep learning image features with clinical data significantly improved the prediction of good reperfusion. The visualization of prediction feature importance showed both known and novel clinical and imaging features with predictive values.

## Introduction

Approximately one-third of patients who suffer from acute ischemic stroke die or become functionally dependent, making stroke a very severe condition worldwide (1). An occlusion in one of the major cerebral arteries is present in one-third of patients, and is often referred as large vessel occlusion (LVO) (2). Endovascular treatment (EVT) is the standard treatment for LVO, and its great benefits have been proven extensively (3–6). However, despite successful treatment, ~30% of patients still present a poor outcome at 3 months. The outcome after treatment is dependent on multiple factors, from patient characteristics and condition to severity and location of the occlusion (7). Accurate prediction of patient outcome has been explored in several studies and it is of utmost importance to correctly identify patients who will and who will not have a good outcome after EVT. This information can be used to further personalize acute stroke care (7, 8).

Most prediction models found in literature focused on the small subsets of clinical features (7), although some recent studies have explored a broader set of variables (8, 9). In most cases, information in radiological images was included in the form of visual scores, such as Alberta stroke program early CT score (ASPECTS) and the collateral score. However, translating millions of voxels in a radiological image to one or several visual scores with a limited number of categories can potentially result in significant information loss.

In this study we explored a more extensive feature representation of radiological images, such as a multitude of handcrafted features (radiomics) and automatically learned features using deep learning approaches. We hypothesized that a more extensive image feature representation can lead to an improved outcome prediction of patients with ischemic stroke through leveraging information that is complementary to or more detailed than radiological scores. On the other hand, a deep learning approach allows the extraction of images features that are automatically learned by the network, reducing the risk of bias. We performed the combination of automatically extracted images features with patient data available at baseline, and evaluated their impact on the prediction accuracy of both clinical and radiological outcomes. Finally, we presented the feature importance for the best models and their impact in the predictions.

## Methods

### Study Population

We included 3,279 patients from the MR CLEAN Registry, which is a prospective, observational, multicenter study, that consecutively included all EVT-treated patients with acute ischemic stroke in the Netherlands since the completion of the MR CLEAN trial in March 2014 (10). The central medical ethics committee of the Erasmus Medical Center Rotterdam, the Netherlands, evaluated the study protocol and granted permission (MEC-2014–235) to carry out the data collection as a registry (11). Patients provided permission for study participation through an opt-out procedure. Data that have been used for this study are available upon reasonable request from the MR CLEAN Registry committee ( mrclean@erasmusmc.nl).

### Variables and Outcome

All radiological and clinical variables available at baseline were included in the models. In total, 50 variables were selectively included. Ordinal variables, such as pre-stroke modified Rankin Scale (mRS), collaterals, ASPECTS, National Institutes of Health Stroke Scale (NIHSS), Clot Burden Score (CBS), and Glasgow Coma Scale were treated as linear continuous scores. For non-ordinal variables with multiple categories, we created separate binary variables for all categories. The final input size for the models consisted of 58 features. A complete list of variables available can be found in Table 1.

**Table 1:** Details of included variables.

**Name**		**Occurrence (%) *N* = 3,279**	**Missing** ***n* (%)**	**Analyzed** **as**
Previous stroke			27 (1)	Cat
	0—no	2,706 (83)		
	1—yes	546 (17)		
Myocardial infarction			67 (2)	Cat
	0—no	2,759 (84)		
	1—yes	453 (14)		
Peripheral arterial disease			68 (2)	Cat
	0—no	2.910 (89)		
	1—yes	301 (9)		
Diabetes			24 (1)	Cat
	0—no	2,723 (83)		
	1—yes	532 (16)		
Hypertension			66 (2)	Cat
	1—yes	1,688 (51)		
	0—no	1,525 (47)		
Atrial fibrillation			43 (1)	Cat
	0—no	2,464 (75)		
	1—yes	772 (24)		
Hypercholesterolemia			143 (4)	Cat
	0—no	2,169 (66)		
	1—yes	967 (29)		
Antiplatelet use			41 (1)	Cat
	0—no	2,227 (68)		
	1—yes	1,011 (31)		
DOAC use			40 (1)	Cat
	0—no	3,132 (96)		
	1—yes	107 (3)		
Coumarin use			24 (1)	Cat
	0—no	2,839 (87)		
	1—yes	416 (13)		
Heparin use			43 (1)	Cat
	0—no	3,135 (96)		
	1—yes	101 (3)		
Blood pressure medication			62 (2)	Cat
	1—yes	1,739 (53)		
	0—no	1,478 (45)		
Statin use			74 (2)	Cat
	0—no	2,070 (63)		
	1—yes	1,135 (35)		
HAS on baseline NCCT			131 (4)	Cat
	1—yes	1,704 (52)		
	0—no	1,444 (44)		
Relevant (new) ischemia / hypodensity			157 (5)	Cat
	1—yes	1,908 (58)		
	0—no	1,214 (37)		
Hemorrhagic transformation			137 (4)	Cat
	0—no	3,098 (94)		
	1—yes	44 (1)		
Leukoariosis			128 (4)	Cat
	0—no	1,903 (58)		
	1—yes	1,248 (38)		
Old infarcts in same ASPECTS region?			126 (4)	Cat
	0—no	2,721 (83)		
	1—yes	432 (13)		
Intracranial atherosclerosis on CTA scored by core lab			132 (4)	Cat
	1—yes	1,886 (58)		
	0—no	1,261 (38)		
Sex			0 (0)	Cat
	Male	1,696 (52)		
	Female	1,583 (48)		
Most proximal occlusion segment on CTA scored by core lab, based on CBS			151 (5)	Cat
	Distal M1	1,061 (32)		
	Proximal M1	754 (23)		
	ICA-T	663 (20)		
	M2	455 (14)		
	Intracranial ICA	161 (5)		
	None	13 (0)		
	M3	9 (0)		
	A2	6 (0)		
	A1	6 (0)		
Smoking			758 (23)	Cat
	0—no	1,813 (55)		
	1—yes	708 (22)		
Inclusion on weekday or weekend			0 (0)	Cat
	0—weekday	2,415 (74)		
	1—weekend	864 (26)		
Admission between 17.00 and 08-00 (weekday)/ weekend or holiday. Based on ER time.			0 (0)	Cat
	1—office hours	2,088 (64)		
	0—outside office hours	1,191 (36)		
Transfer from other hospital			1 (0)	Cat
	1—transfer	1,783 (54)		
	0—no transfer	1,495 (46)		
Contraindications for IVT			2,461 (75)	Cat
	0—no	772 (24)		
	1—yes	46 (1)		
No abnormalities at symptomatic carotid bifurcation on CTA baseline by core lab			400 (12)	Cat
	0—no abnormalities	2,110 (64)		
	1—any abnormalities	769 (23)		
50% or more atherosclerotic stenosis at symptomatic carotid bifurcation on CTA baseline			400 (12)	Cat
	0—no	2,615 (80)		
	1—yes	264 (8)		
Atherosclerotic occlusion at symptomatic carotid bifurcation on CTA baseline by core lab			400 (12)	Cat
	0—no	2,564 (78)		
	1—yes	315 (10)		
Floating thrombus at symptomatic carotid bifurcation on CTA baseline by core lab			400 (12)	Cat
	0—no	2,826 (86)		
	1—yes	53 (2)		
Pseudo-occlusion at symptomatic carotid bifurcation on CTA baseline by core lab			400 (12)	Cat
	0—no	2,684 (82)		
	1—yes	195 (6)		
Carotid dissection at symptomatic carotid bifurcation on CTA baseline by core lab			400 (12)	Cat
	0—no	2,777 (85)		
	1—yes	102 (3)		
Occlusion side on CTA scored by core lab			2 (0)	Cat
	Left hemisphere	1,745 (53)		
	Right hemisphere	1,515 (46)		
	Neither	17 (1)		
In-hospital stroke			534 (16)	Cat
	0—no	2,416 (74)		
	1—yes	329 (10)		
Second occlusion in other territory present on CTA scored by core lab			546 (17)	Cat
	0—no	2,454 (75)		
	1—yes	279 (9)		
Collateral score on CTA scored by core lab			207 (6)	Cont
	100% of occluded area	595 (18)		
	>50% but less <100%	1,190 (36)		
	filling <50% of occluded area	1,100 (34)		
	Absent collaterals	187 (6)		
Pre-stroke mRS			72 (2)	Cont
	0	2,170 (66)		
	1	424 (13)		
	2	241 (7)		
	3	211 (6)		
	4	133 (4)		
	5	28 (1)		
90-day mRS			214 (7)	Cat
	6	886 (27)		
	2	561 (17)		
	1	471 (14)		
	3	404 (12)		
	4	366 (11)		
	0	209 (6)		
	5	168 (5)		
Post-eTICI			90 (3)	Cat
	3	905 (28)		
	2b	702 (21)		
	2a	597 (18)		
	0	543 (17)		
	2c	347 (11)		
	1	95 (3)		
ASPECTS baseline scored by core lab—median (IQR)		9 (7–10)	109 (3)	Cont
CBS at baseline—median (IQR)		6 (4–8)	766 (23)	Cont
NIHSS at baseline—median (IQR)		16 (11–20)	55 (2)	Cont
Glucose level at baseline—median (IQR)		7 (6–8)	371 (11)	Cont
RR systolic at baseline—median (IQR)		150 (131–165)	89 (3)	Cont
RR diastolic at baseline—median (IQR)		80 (71–91)	97 (3)	Cont
INR at baseline—median (IQR)		1 (1–1)	608 (19)	Cont
Thrombocyte count at baseline—median (IQR)		234 (194–289)	445 (14)	Cont
CRP level at baseline—median (IQR)		4 (2–10)	651 (20)	Cont
Age—median (IQR)		72 (61–80)	0 (0)	Cont
Total glasgow coma scale at baseline—median (IQR)		13 (11–15)	113 (3)	Cont
Duration from onset to groin in minutes—median (IQR)		195 (150–260)	15 (0)	Cont
Duration: onset to IVT in minutes in first hospital—median (IQR)		24 (18–33)	1,353 (41)	Cont

*A1, first segment of anterior cerebral artery; ASPECTS, Alberta stroke program early CT score; cat, categorical; CBS, clot burden score; cont, continuous; CRP, C-reactive protein; CTA, CT angiography; DOAC, direct oral anticoagulant; ER, emergency room; HAS, hyperdense artery sign; IQR, interquartile range; M1/M2/M3, first/second/third segment of middle cerebral artery; mRS, modified Rankin Scale; NCCT, non-contrast CT; NIHSS, National Institutes of Health stroke scale; RR, blood pressure (Riva-Rocci)*.

We created the two sets of prediction models for the following outcome variables, (1) favorable functional outcome after 3 months, defined by the mRS ≤ 2 and (2) good reperfusion defined by the modified Thrombolysis in Cerebral Infarction (eTICI)-score after EVT (post-eTICI ≥ 2b).

### Image Data Pre-Processing

We included CT angiography (CTA) scans from all patients available in the dataset, following the approach from (12), where the added value of CTA for outcome predictions has already been proven. The first step in pre-processing the images was to strip the skull, since it contains voxels that are not relevant for the prediction tasks (13). For this segmentation task, we used a U-Net (14), (a convolutional neural network designed for the segmentation of biomedical images) trained on skull segmentations that were created using the approach described in (15) and subsequently manually corrected.

Since the slice thickness and the orientation of the head varied significantly between different scans, we registered the images to a reference scan using rigid and affine transformations. For this, we used an atlas as a reference scan (16), with a size of 256 × 256 × 90 voxels. This atlas was developed using the Laboratory of Neuro Imaging Probabilistic Brain Atlas (LPBA40), which is publicly available (17). The atlas served not only as a reference for registration, but it also contains the annotation of 70 brain regions, which allowed the region-based feature extraction.

### Radiomics Approach

For the first approach, we computed radiomics features for each of the 70 brain regions of the scans that were registered to the atlas. An advantage of crafting features from specific regions is that we can easily trace back which regions of the brain were the most important for prediction. We used all 70 regions contained in the atlas. For each region, 18 first-order features (Supplementary Table I) were computed using the Pyradiomics library (18). This resulted in a total of 1,260 features. Since some regions overlapped and others were relatively small, we checked the correlation between the features using the Pearson correlation coefficient. Due to the large number of correlated features, we only kept the ones that were <50% correlated to the others (19) reducing the number of radiomics features to 68. This was necessary since multiple highly correlated features (multicollinearity) can hamper learning (19). For example, in the case of Logistic Regression (LR), multicollinearity can lead severe variations in the coefficients, making the results less robust and trustworthy. Moreover, the large number of features would also be a problem because of the limited sample size *n* = 3,001 (20). A complete list of the computed features is presented in the Supplementary Table I. These features were subsequently combined (concatenated) with the clinical data available at baseline and used to create prediction models for the outcomes. We selected the following state-of-the-art machine learning techniques from different families: random forest classifier (RFC) (21), support vector machine (SVM) (22), artificial neural networks (NN) (23), gradient boosting (XGB) (24), and logistic regression (LR).

### Deep Learning Approach

In the deep learning approach, the skull-stripped scans were used to train convolutional neural networks (CNNs) to predict the outcome. We opted for using skull-stripped scans prior to the image registration to keep the information in the scans as raw as possible, and avoid changes in the Hounsfield units caused by the registration process. Moreover, we included transformations for data augmentation, such as rotation and flipping, which makes registration a futile step. Therefore, the registered scans were only used in *radiomics* approach. Since the input for deep learning models has to be uniform, and the voxel size among scans was not, we resampled the scans in all directions. The final input size to the network was 256 × 256 × 30 voxels. To increase the number of training samples and account for possible variations in the data, we performed data augmentation (vertical flipping and rotation) using the training set. We trained and optimized 3D CNNs to predict the favorable functional outcome and good reperfusion.

We selected the ResNet10 architecture since it has produced comparable results to the deeper architectures in medical imaging related tasks (25) while keeping training time feasible. For each network implemented, we added a Squeeze and Excitation (SE) module before the fully-connected layers (26), since it has been shown to greatly improve the results in diverse ResNet models in multiple prediction tasks. The SE module models interdependencies between channels by adding learnable weights channels-wise. This way, the contribution of certain features in a given channel can have more or less impact than the others in the final prediction. Finally, we combined the clinical features with the image features by concatenating the clinical features to the image features before the fully-connected layers of the CNN (27–29).

The models were trained from scratch with the SE module. Finally, we explored the effect of Transfer Learning in our models, leveraging the model developed in (25). In that model, the aim was to develop a robust ResNet by training it in a large amount of medical data (by putting together multiple datasets), such as CT and MRI scans. The resulting CNN was able to learn filters that can extract relevant image features and generalized well to other tasks, making it ideal for transfer learning to other datasets with less images.

The ResNet model was trained from scratch for 75 epochs, and for 50 epochs when Transfer Learning was used. Following the results presented in (30), we opted to train the models built from scratch for longer than those using Transfer Learning to allow for a more fair comparison. We optimize our models using the Focal Loss (31), since there was some class imbalance in our labels (~0.4/0.6 for both labels). Finally, we used an Adam optimizer (32), with a learning rate of 0.001 and the weight decay to 0.00006. The other hyper-parameters were left unchanged. The mini-batch size was kept at two due to memory limitations.

### Pipeline and Experimental Setup

Several of the 58 clinical variables included in our experiments had missing values. We imputed the missing values (from the features and outcomes) using Multiple Imputation with Chained Equations (33), since it has shown the state-of-the-art results. Imputation was performed on the training and test sets separately, by training the imputation model on the training set and applying it to the test set to prevent the data leakage. The data were then scaled by subtracting the mean and divided by the standard deviation (SD), for the optimal performance of ML models. We used an inner and outer k-fold cross-validation strategy (nested cross-validation) to train, validate, and test our models. First, in the outer cross-validation loop, the dataset was split into training and testing using a 5-fold cross-validation strategy (4-folds are used for training and 1 for testing). The training set was then split again (inner-cross-validation) into training and validation, with 20% being used for validation. The validation set is used to assess the model performance during training, for early-stopping and for hyper-parameter optimization. All experiments (based on radiomics or deep learning) used the same cross-validation setup described above. The list of the hyper-parameters used to optimize the models is shown in the Supplementary Table II. The assessment and final reporting of performance was done on the test sets.

For each approach, we designed four experiments to predict the outcomes: first (coined *clinical*), using all clinical features available at baseline, therefore including patient demographics and image-derived scores, such as ASPECTS; second (coined *image*), using only the features hand-crafted or learned (radiomics or deep learning features) from the CTA scans; third (coined *combination*), by combining all the features from the first experiment with the features of the second (all clinical data available at baseline, such as image scores, and features learned from CTA scans), and fourth (coined *no image score*), by repeating the first and third experiments, but without any image derived scores, such as ASPECTS or collateral scores.

We evaluated the models using the area under the receiver operating characteristic (ROC) curve (AUC), the negative predictive value (NPV), the positive predictive value (PPV), the sensitivity, and specificity. To assess statistically significant differences between the models, we reported the confidence intervals (*CI*s) for all the cross-validation iterations and used McNemar's and Delong's tests (34, 35).

**Figure 1:**
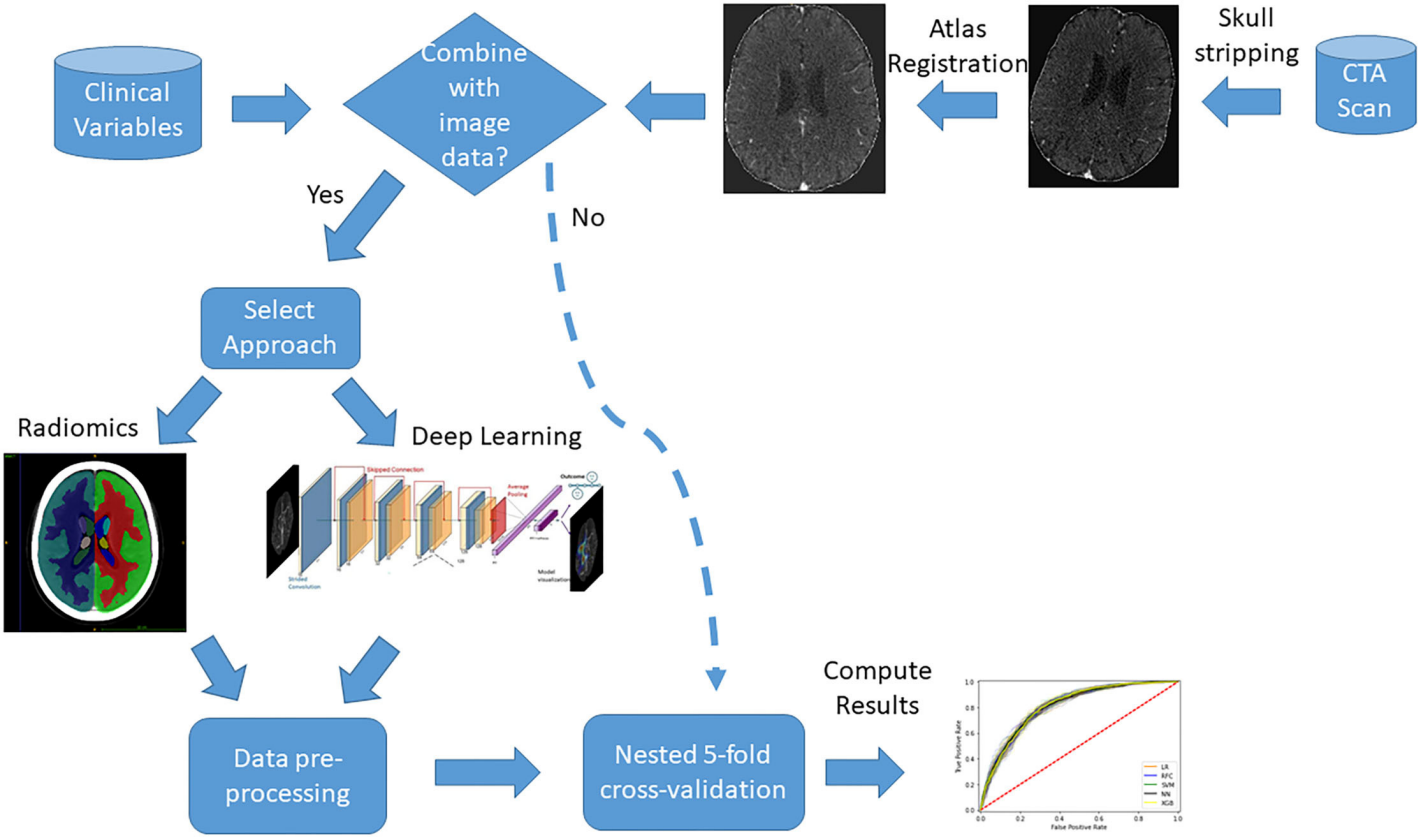
Diagram with an overview of the main data pre-processing steps and combination approaches.

A diagram is presented in Figure 1 with an overview of the main imaging pre-processing steps and data combination approaches.

All codes used for the development of models and data analysis are available at: https://github.com/L-Ramos/mrclean_combination.

### Feature Importance

To visualize the feature importance in our models, we used SHapley Additive exPlanations (SHAP), which is based on a game theory to explain the output of any machine learning model (36). In SHAP, the contribution of a feature is given by computing the average contribution of all features by permuting all of them. SHAP has many advantages over other feature visualization techniques, such as Local Interpretable Model-agnostic Explanations (LIME) (37). SHAP provides global explanations instead of sample-oriented ones, offers tools to evaluate the feature dependence and interactions and, the output explanations are generated based on the trained model provided by the user, instead of training a new model to explain the feature importance, which is the case for LIME. Finally, with SHAP, the impact of low and high values of a given feature in the final outcome can be more clearly evaluated with the plots, along with how important the feature is in predicting the correct class (36). Positive SHAP values (above zero in the *x*-axis) mean that the feature values are associated to the positive class (good functional outcome or reperfusion), while SHAP values below zero indicate the opposite.

## Results

### Study Population

**Figure 2:**
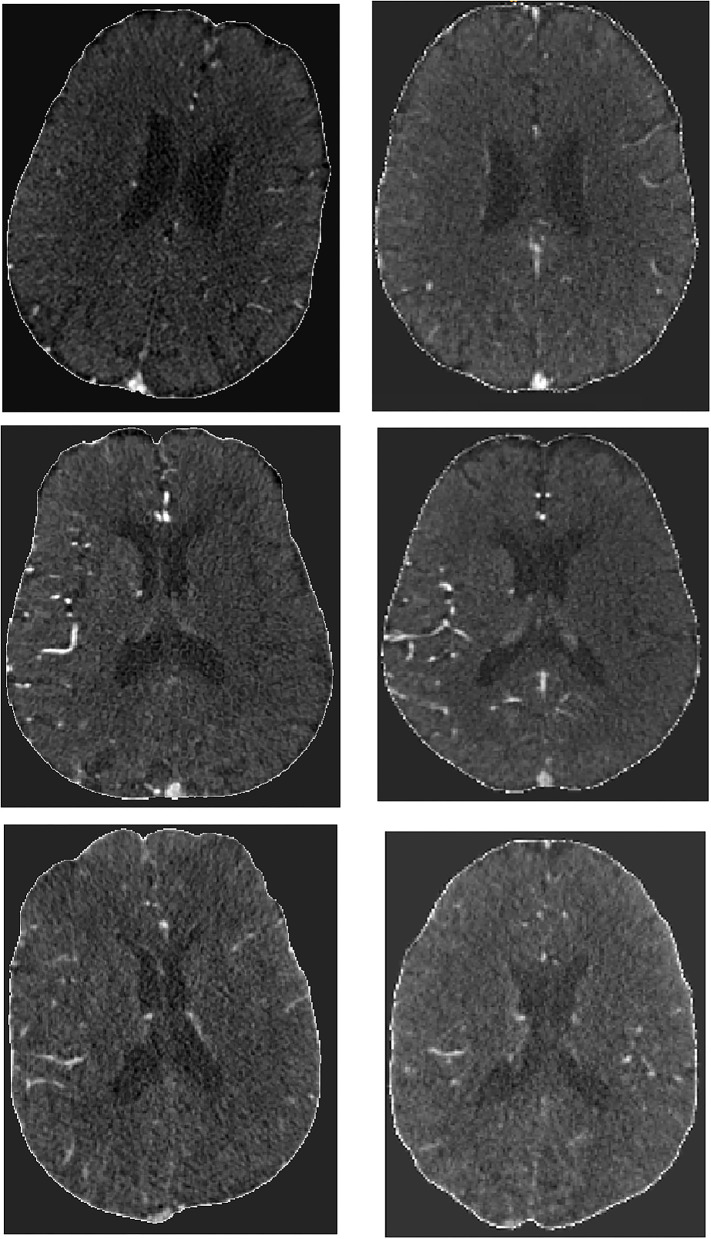
Example of skull stripped scans (Left) and post-registration results (Right).

Of 3,279 patients that were eligible for this study, 278 were excluded due to either failure during skull-stripping (133 patients), or because of incomplete scans, severe artifacts or due to failure during image registration (145 patients). In total, 3,001 patients were included, the mean age was 72 years old, and median baseline NIHSS was 16 (Table 1). At 90 days, 1,241 (37%) patients had a good functional outcome (mRS ≤ 2) with 214 missing values (7%). Regarding reperfusion (post-eTICI ≥ 2b), 1,954 (60%) patient had good reperfusion after treatment, with 90 missing values (3%). Figure 2 contains a few examples of skull-stripped scans (on the left) and a few examples of scans that were registered to the atlas (on the right). It is important to highlight that due to registration, the scans no longer have the same slice thickness and number of slices, so there is no one-to-one matching.

### Radiomics Approach

We present the results of good functional outcome prediction using the *radiomics* approach in Table 2. The AUC value was the highest for the *clinical* experiment (0.81), though it does not differ significantly from the *combination* experiment. The AUC is the lowest for the *image* experiment (0.69). For the *combination* experiment, the best AUC was 0.80. Sensitivity was the highest for the clinical experiment (0.79), while specificity was the highest for the *combination* (0.77). The difference between the *clinical* and the *combination* experiments was not statistically significant (*p* = 0.12) for the RFC.

**Table 2:** Results of the clinical, image, and combination for the radiomics approach for predicting the good functional outcome (mRS ≤ 2).

**Methods**	**AUC 95% CI**	**F1-Score**	**Sensitivity**	**Specificity**	**PPV**	**NPV**
**Clinical experiment**						
RFC	0.81 (0.79–0.82)	0.69 (0.67–0.72)	0.72 (0.68–0.76)	**0.75 (0.72–0.77)**	0.67 (0.63–0.71)	0.79 (0.76–0.82)
SVM	0.81 (0.80–0.83)	0.71 (0.68–0.74)	**0.79 (0.75–0.82)**	0.70 (0.68–0.72)	0.65 (0.61–0.69)	0.82 (0.79–0.85)
LR	0.81 (0.80–0.82)	0.71 (0.68–0.73)	0.77 (0.74–0.80)	0.71 (0.69–0.73)	0.65 (0.62–0.69)	0.81 (0.78–0.84)
XGB	0.81 (0.80–0.82)	0.71 (0.68–0.74)	0.77 (0.74–0.81)	0.71 (0.70–0.72)	0.66 (0.62–0.69)	0.82 (0.79–0.84)
NN	0.81 (0.80–0.82)	0.69 (0.68–0.71)	0.73 (0.66–0.80)	0.74 (0.67–0.81)	0.67 (0.60–0.73)	0.79 (0.75–0.84)
**Image experiment**						
RFC	0.68 (0.65–0.70)	0.50 (0.42–0.58)	0.45 (0.33–0.57)	**0.77 (0.71–0.83)**	0.58 (0.53–0.62)	0.66 (0.61–0.71)
SVM	0.69 (0.66–0.71)	0.60 (0.54–0.65)	**0.64 (0.58–0.71)**	0.64 (0.62–0.66)	0.56 (0.50–0.62)	0.72 (0.67–0.76)
LR	0.68 (0.66–0.70)	0.58 (0.53–0.63)	0.60 (0.53–0.66)	0.67 (0.65–0.69)	0.56 (0.53–0.60)	0.70 (0.65–0.74)
XGB	0.67 (0.65–0.69)	0.55 (0.52–0.58)	0.56 (0.51–0.61)	0.67 (0.63–0.71)	0.55 (0.51–0.59)	0.68 (0.64–0.72)
NN	0.65 (0.59–0.71)	0.49 (0.45–0.52)	0.45 (0.37–0.52)	0.72 (0.61–0.83)	0.54 (0.48–0.61)	0.65 (0.60–0.69)
**Combination experiment**						
RFC	0.80 (0.79–0.81)	0.67 (0.64–0.70)	0.66 (0.60–0.73)	**0.77 (0.72–0.82)**	0.67 (0.63–0.72)	0.76 (0.73–0.80)
SVM	0.79 (0.78–0.81)	0.70 (0.67–0.73)	**0.78 (0.73–0.82)**	0.68 (0.66–0.71)	0.64 (0.60–0.67)	0.81 (0.78–0.84)
LR	0.80 (0.78–0.81)	0.70 (0.66–0.73)	0.76 (0.72–0.80)	0.70 (0.68–0.73)	0.65 (0.60–0.69)	0.80 (0.78–0.83)
XGB	0.80 (0.78–0.81)	0.69 (0.67–0.71)	0.76 (0.72–0.79)	0.69 (0.66–0.72)	0.64 (0.61–0.67)	0.80 (0.77–0.83)
NN	0.78 (0.77–0.79)	0.67 (0.65–0.68)	0.64 (0.60–0.68)	0.74 (0.70–0.75)	0.66 (0.62–0.68)	0.74 (0.68–0.76)

*Average over 5-fold cross-validation. RFC, random forest classifier; SVM, support vector machine; LR, logistic regression; XGB, gradient boosting; NN, neural networks. AUC, area under the curve; NPV, negative predictive value; PPV, positive predictive value. Values in bold indicate the best Sensitivity and Specificity values for a given experimental setup*.

There was no significant difference (drop of 0.01 or at most 0.02 in the average of all measures) in the results of the *no image score* experiment when compared with other experiments as shown in Supplementary Table III.

In Table 3, we show the results for good reperfusion prediction (post-eTICI ≥ 2b). The highest AUC was for the *combination* experiment (0.57), while the lowest was for the clinical experiment (0.51). Sensitivity was the highest (0.91) for the image experiment, but specificity was the lowest (0.11), showing that the RFC model might be biased toward one of the classes in this experiment. The same does not occur for all models in the image experiment, LR for instance, shows a good balance between sensitivity and specificity values for all experiments. The difference between the *clinical* and the *combination* experiments was statistically significant, *p* = 0.008 for the RFC. We provide results from the Delong test in **Table 6**, which confirmed that the difference between the *clinical* and *combinations* experiments was statistically significant.

**Table 3:** Results of the clinical, image, and combination experiments for the radiomics approach for predicting the good reperfusion [post-modified Thrombolysis in Cerebral Infarction (eTICI) ≥ 2b].

**Methods**	**AUC 95% CI**	**F1-Score**	**Sensitivity**	**Specificity**	**PPV**	**NPV**
**Clinical experiment**						
RFC	0.53 (0.51–0.55)	0.71 (0.68–0.74)	0.79 (0.74–0.84)	0.26 (0.19–0.32)	0.64 (0.60–0.69)	0.42 (0.36–0.48)
SVM	0.54 (0.53–0.56)	0.39 (0.08–0.70)	0.32 (0.01–0.64)	0.73 (0.44–1.02)	0.68 (0.65–0.72)	0.39 (0.35–0.43)
LR	0.54 (0.51–0.56)	0.61 (0.57–0.66)	0.59 (0.54–0.64)	0.44 (0.39–0.50)	0.64 (0.61–0.68)	0.39 (0.34–0.43)
XGB	0.51 (0.50–0.54)	0.63 (0.57–0.69)	0.63 (0.55–0.71)	0.37 (0.30–0.45)	0.63 (0.58–0.68)	0.37 (0.33–0.41)
NN	0.51 (0.50–0.53)	0.70 (0.62–0.79)	0.81 (0.60–1.03)	0.19 (0.03–0.41)	0.63 (0.59–0.67)	0.37 (0.32–0.43)
**Image experiment**						
RFC	0.54 (0.52–0.56)	0.75 (0.74–0.75)	0.91 (0.81–1.01)	0.11 (0.01–0.22)	0.64 (0.59–0.68)	0.42 (0.35–0.50)
SVM	0.55 (0.53–0.57)	0.70 (0.61–0.79)	0.79 (0.55–1.03)	0.25 (0.03–0.53)	0.64 (0.60–0.69)	0.41 (0.37–0.46)
LR	0.53 (0.50–0.57)	0.61 (0.57–0.64)	0.57 (0.53–0.61)	0.47 (0.40–0.54)	0.65 (0.61–0.69)	0.39 (0.32–0.46)
XGB	0.53 (0.50–0.56)	0.64 (0.60–0.68)	0.65 (0.54–0.75)	0.39 (0.28–0.49)	0.64 (0.61–0.68)	0.40 (0.33–0.46)
NN	0.53 (0.50–0.56)	0.67 (0.65–0.69)	0.69 (0.66–0.73)	0.36 (0.32–0.40)	0.65 (0.61–0.69)	0.41 (0.35–0.46)
**Combination experiment**						
RFC	0.57 (0.55–0.59)	0.75 (0.71–0.78)	0.89 (0.85–0.93)	0.15 (0.10–0.19)	0.64 (0.60–0.68)	0.45 (0.38–0.52)
SVM	0.57 (0.54–0.61)	0.63 (0.59–0.66)	0.58 (0.55–0.61)	0.52 (0.46–0.58)	0.68 (0.63–0.72)	0.42 (0.38–0.47)
LR	0.57 (0.54–0.60)	0.63 (0.60–0.66)	0.59 (0.57–0.62)	0.50 (0.46–0.55)	0.67 (0.63–0.72)	0.42 (0.38–0.46)
XGB	0.57 (0.55–0.58)	0.59 (0.55–0.64)	0.54 (0.46–0.61)	0.55 (0.46–0.63)	0.67 (0.63–0.71)	0.41 (0.36–0.46)
NN	0.53 (0.51–0.55)	0.66 (0.62–0.70)	0.68 (0.63–0.72)	0.37 (0.32–0.43)	0.65 (0.60–0.70)	0.40 (0.37–0.43)

*Average over 5-fold cross-validation. RFC, random forest classifier; SVM, support vector machine; LR, logistic regression; XGB, gradient boosting; NN, neural networks. AUC, area under the curve; NPV, negative predictive value; PPV, positive predictive value*.

**Figure 3:**
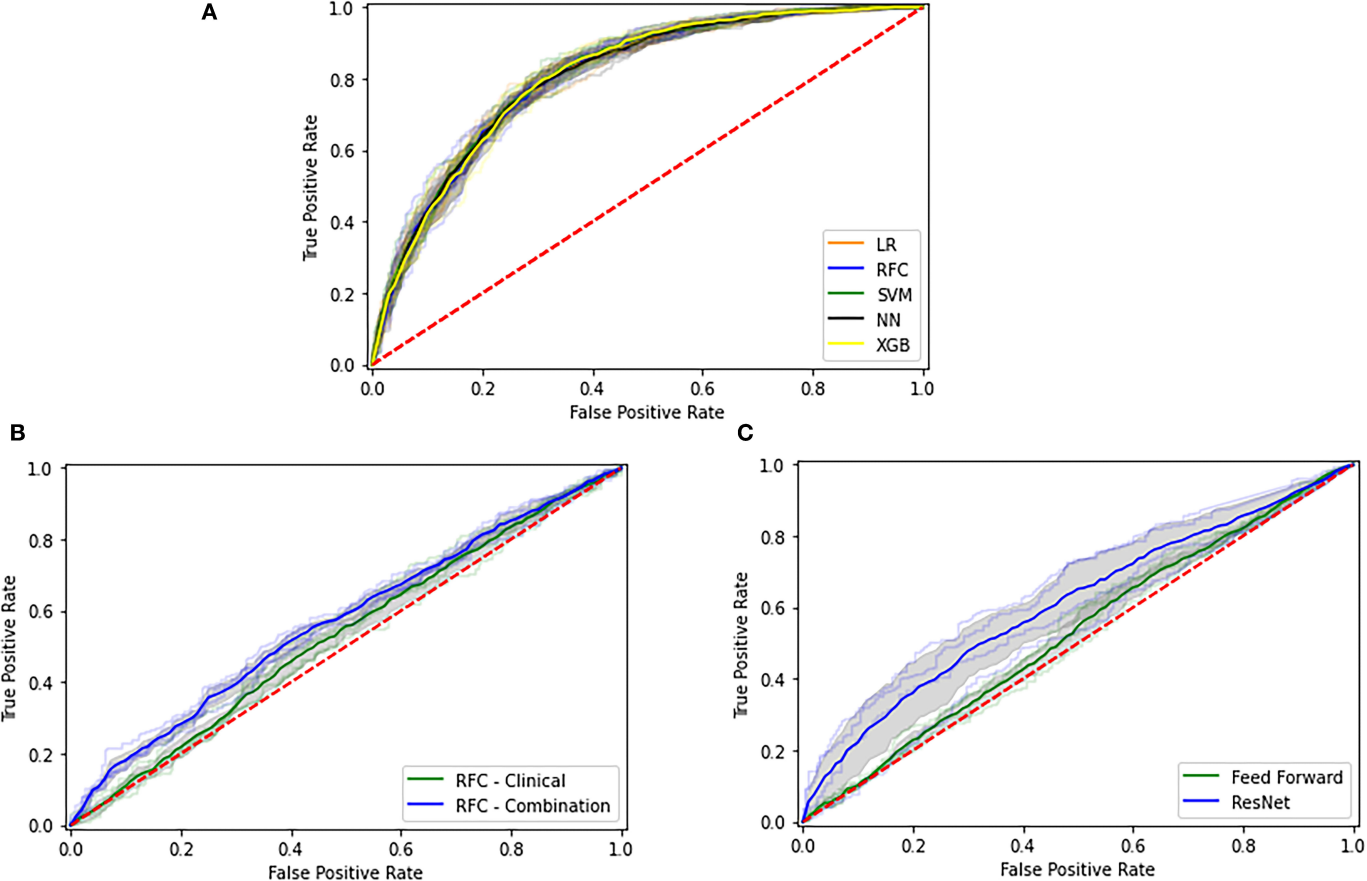
Receiver operating characteristic (ROC) for the different experimental setups. **(A)** The modified Rankin Scale (mRs) prediction for the clinical experiment, **(B)** Modified Thrombolysis in Cerebral Infarction (eTICI) prediction using the radiomics data, and **(C)** eTICI prediction using deep learning.

**Figure 4:**
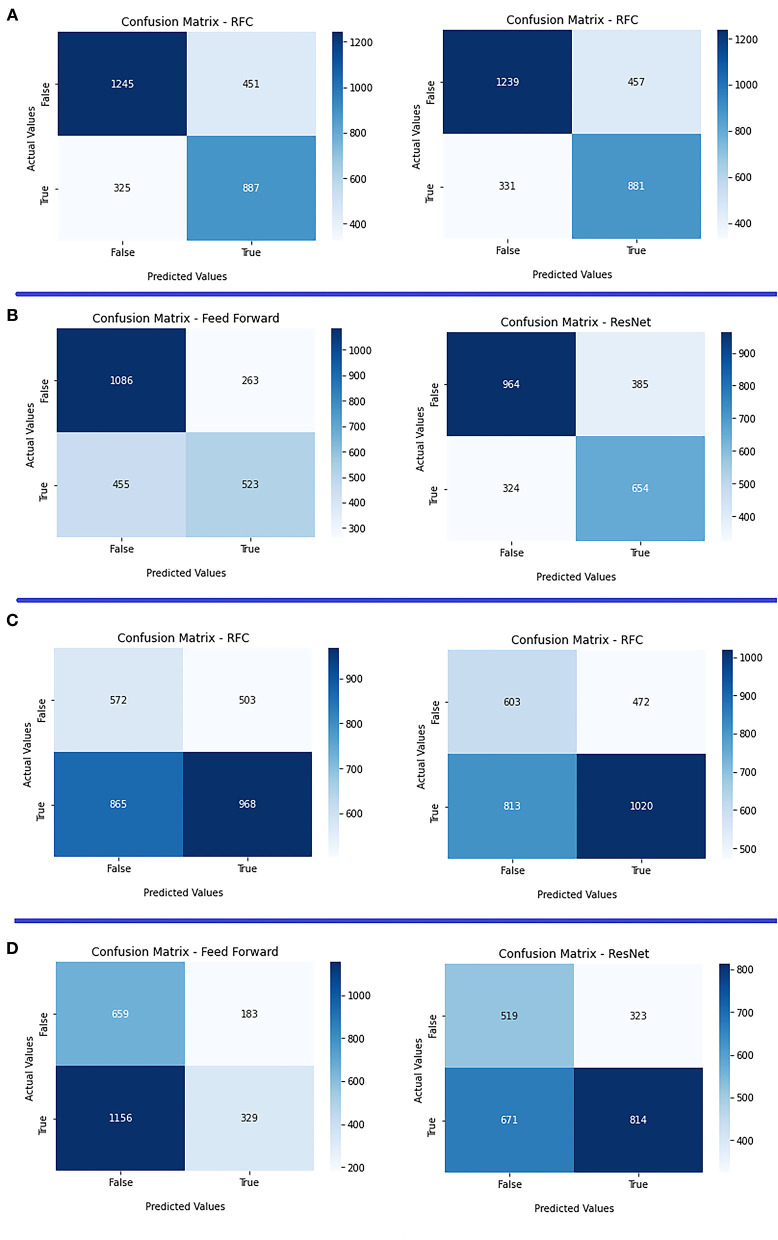
Confusion matrices of both radiomics and deep learning approaches. **(A)** *Clinical* experiment (left) vs. *combination (right)* for mRs prediction using the radiomics approach; **(B)** *clinical* experiment (left) vs. *combination (right)* for mRs prediction using the deep learning approach; **(C)** *clinical* experiment (left) vs. *combination (right)* for eTICI prediction using radiomics approach; and **(D)** *clinical* experiment (left) vs. *combination (right)* for eTICI prediction using the deep learning approach.

We present the ROC curves for all models trained in the *clinical* experiment for predicting mRs in Figure 3A and a comparison between the *clinical* and *combination* experiment for predicting eTICI in Figure 3B. Moreover, we provide the confusion matrices for the *clinical* and *combination* experiments for mRs in Figure 4A and eTICI in Figure 4C.

Finally, there was no significant difference in the measures for the *no image scores* experiment as shown in Supplementary Table IV.

### Feature Importance—Radiomics Approach

**Figure 5:**
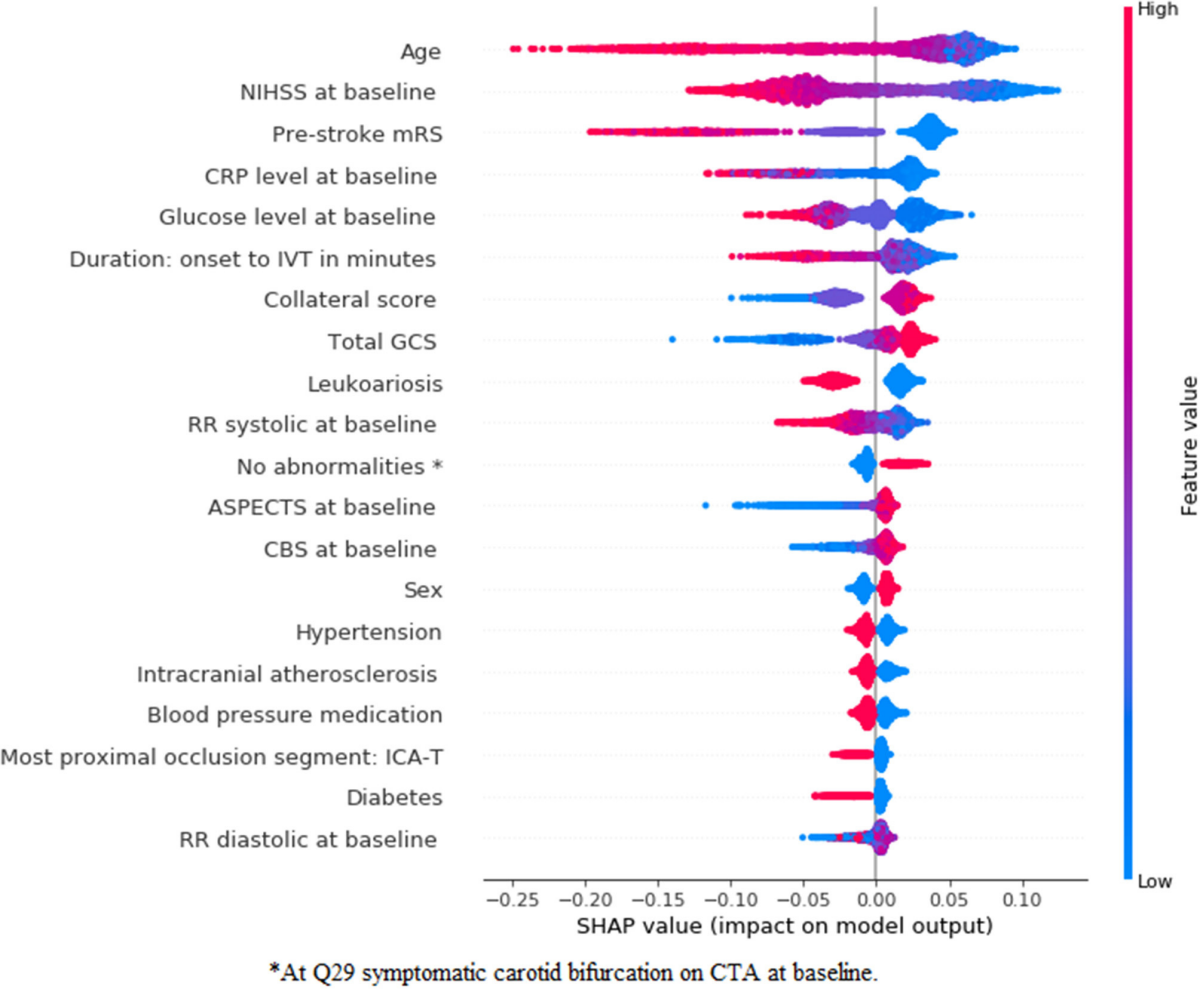
SHapley Additive exPlanations (SHAP) feature importance for the clinical experiment for mRS prediction using the random forest classifier (RFC) model. For visualization purposes, we included only the top 20 features. Features are shown in order of importance, from most important (top) to less important (bottom). The color legend on the right shows how the feature values influence outcome: high values are depicted in red, while low values are presented in blue. Positive SHAP values (above zero in the *x*-axis) mean that the feature values are associated to the positive outcome (in this case good functional outcome), while SHAP values below zero indicate the opposite. ^*^At symptomatic carotid bifurcation on CT angiography (CTA) at baseline.

**Figure 6:**
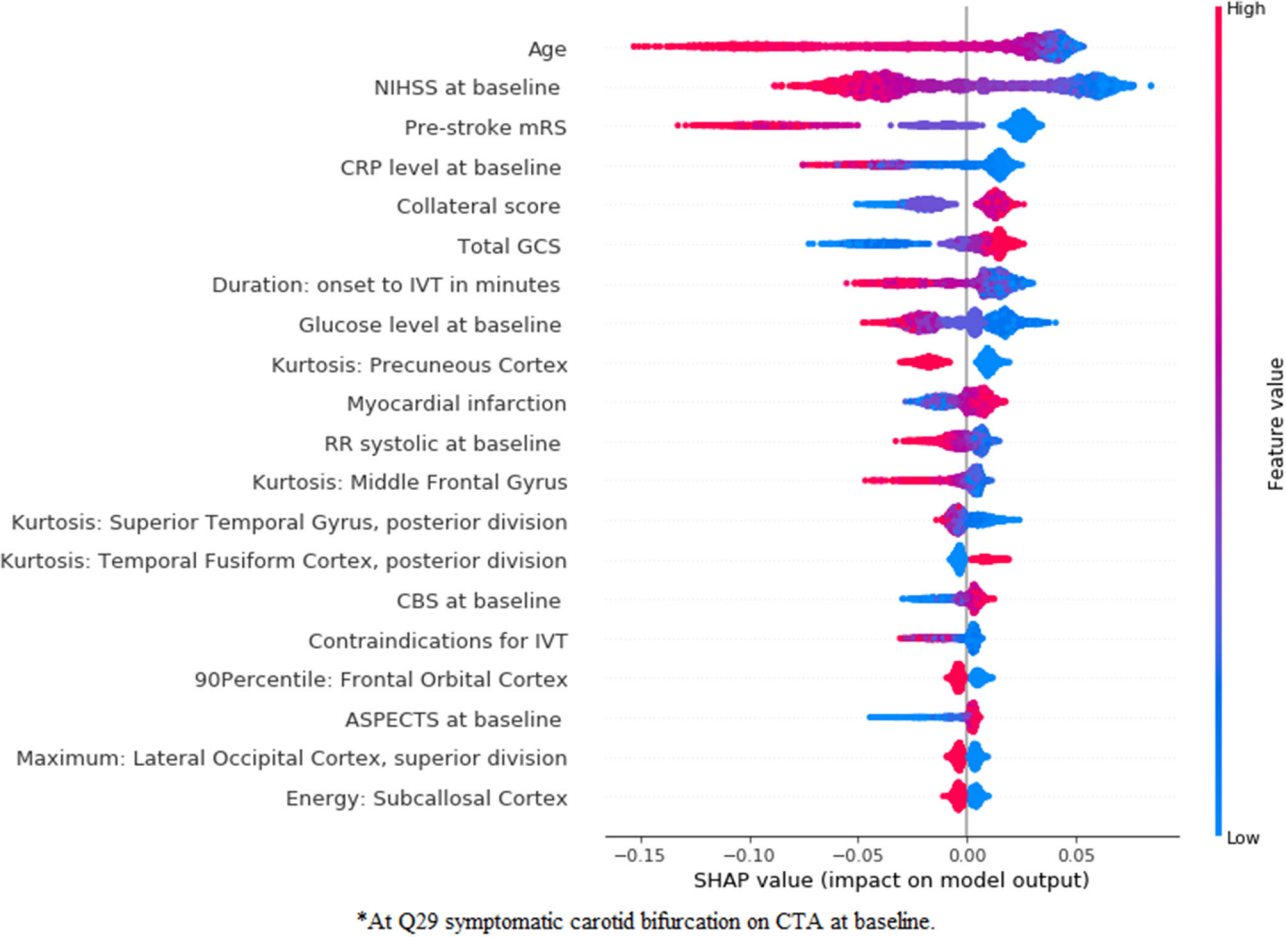
SHapley Additive exPlanations feature importance for the combination experiment for mRS prediction using an RFC model. For visualization purposes, we included only the top 20 features. Features are shown in order of importance, from most important (top) to less important (bottom). The color legend on the right shows how the feature values influence outcome: high values are depicted in red, while low values are presented in blue. Positive SHAP values (above zero in the *x*-axis) mean that the feature values are associated with the positive outcome (in this case good functional outcome), while SHAP values below zero indicate the opposite. ^*^At symptomatic carotid bifurcation on CTA at baseline.

In Figures 5, 6, we present feature importance using SHAP for the *clinical* (Figure 5) and the *combination* (Figure 6) experiments for RFC model and mRS prediction (for all 5-fold cross-validation iterations). We do not present feature importance for the *Image* experiment since results for this experiment were often inferior and in clinical practice, patient demographics are always taken into account for decision-making. The performance measures were the same across the models, therefore, we present feature importance for the RFC model only, since SHAP has extensive support for three-based models. Despite the addition of multiple radiomics features in the *combination* experiment, the top three most important features remain the same for both experiments (age, NIHSS at baseline, and pre-stroke mRS). Ii is clear that the low values of these three features are associated with good functional outcome. Collateral score and the GCS become more important when the radiomics features are combined to the clinical data. Other features, such as leukoariosis and sex seem to lose importance when the radiomics features are combined. Finally, radiomics features from the following regions seem to have a significant impact on the prediction model, despite the lack of improvements in the performance measures: precuneus cortex, middle frontal gyrus, superior temporal gyrus, temporal fusiform cortex, frontal orbital cortex, lateral occipital cortex, and subcallosal cortex.

**Figure 7:**
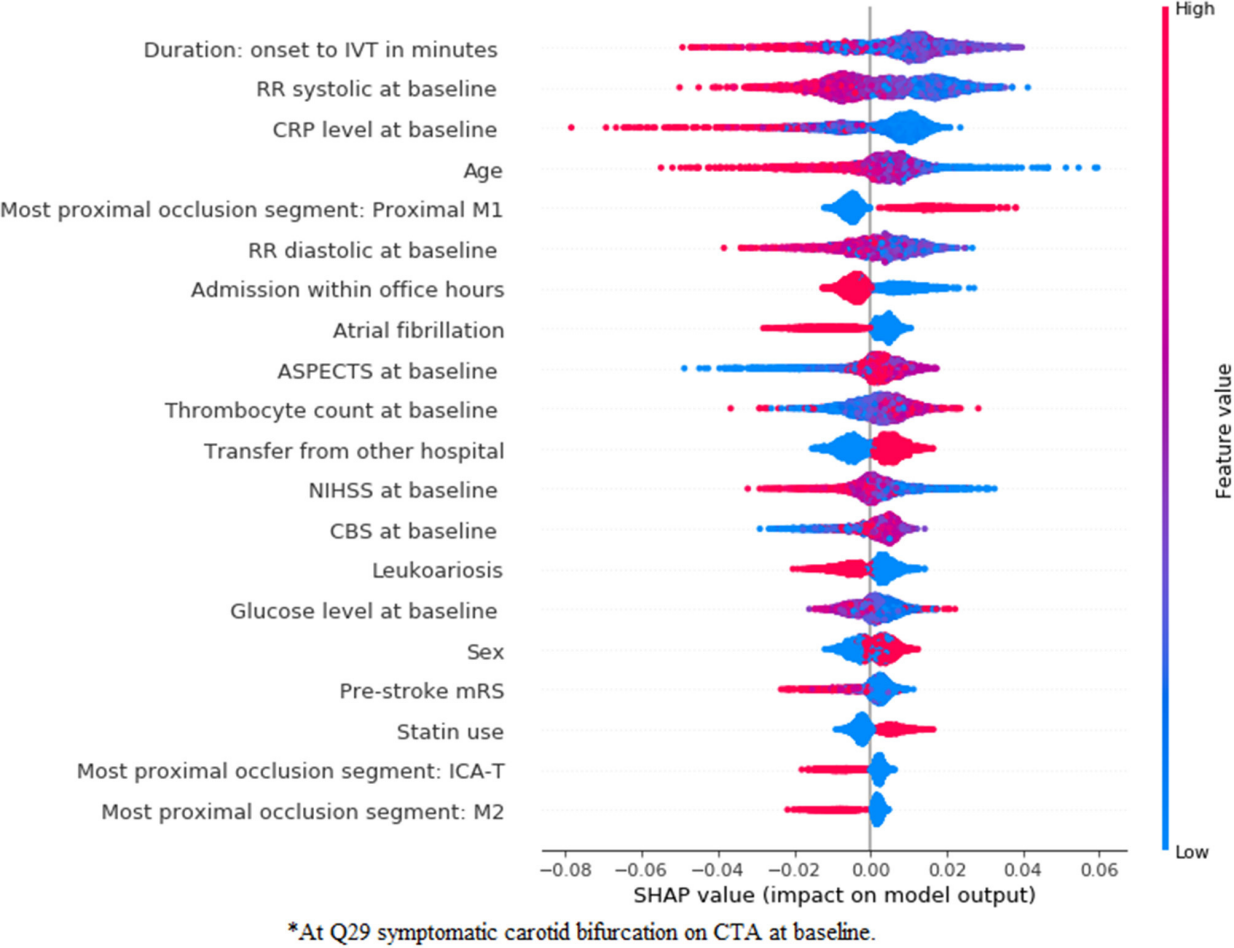
SHapley Additive exPlanations feature importance for the clinical experiment for the prediction of good reperfusion using an RFC model. For visualization purposes we included only the top 20 features. Features are shown in order of importance, from most important (top) to less important (bottom). The color legend on the right shows how the feature values influence outcome: high values are depicted in red, while low values are presented in blue. Positive SHAP values (above zero in the *x*-axis) mean that the feature values are associated to the positive outcome (in this case good r), while SHAP values below zero indicate the opposite. ^*^At symptomatic carotid bifurcation on CTA at baseline.

**Figure 8:**
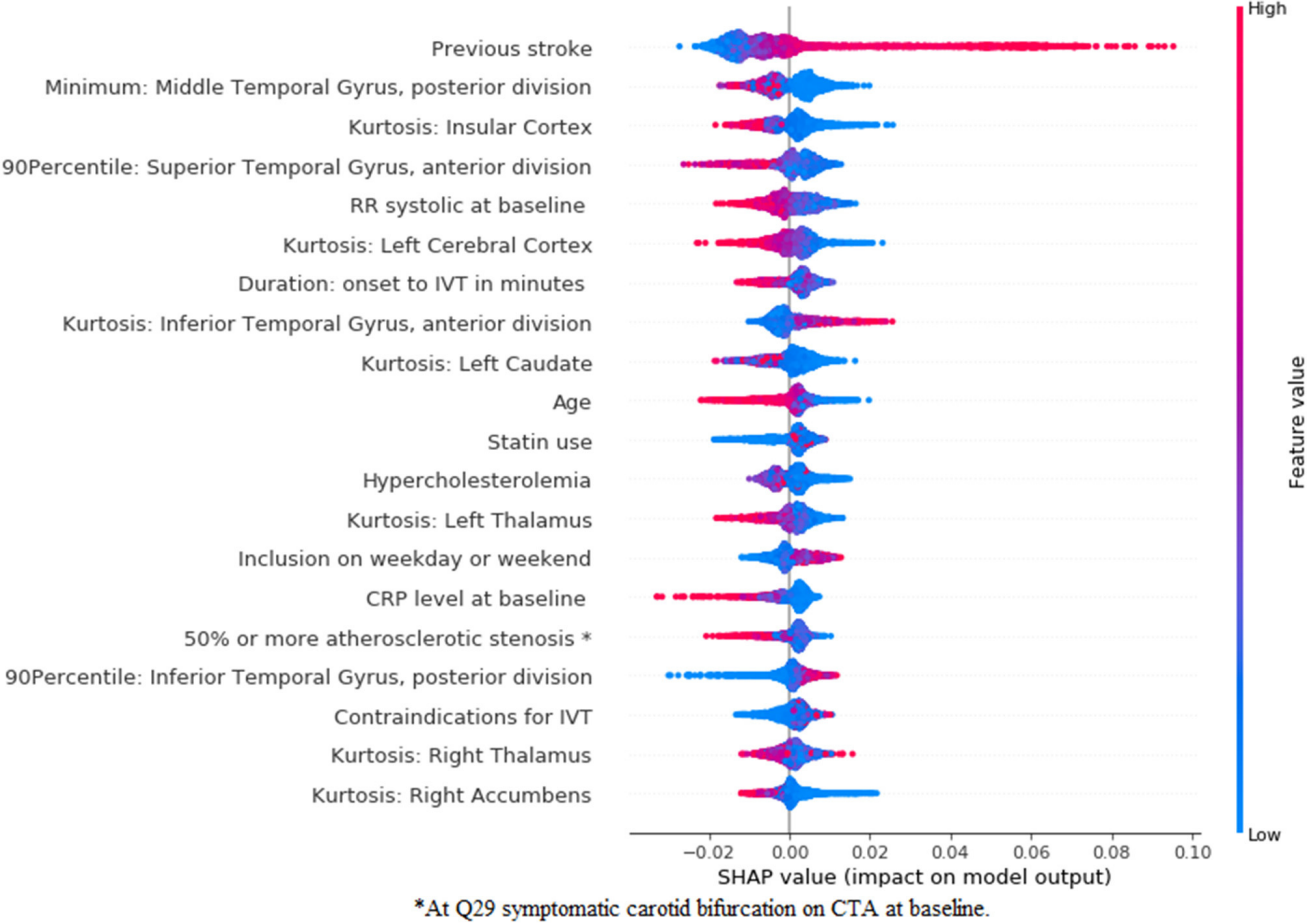
SHapley Additive exPlanations feature importance for the *combination* experiment for the prediction of good reperfusion using an RFC model. For visualization purposes, we included only the top 20 features. Features are shown in order of importance, from most important (top) to less important (bottom). The color legend on the right shows how the feature values influence outcome: high values are depicted in red, while low values are presented in blue. Positive SHAP values (above zero in the *x*-axis) mean that the feature values are associated to the positive outcome (in this case good r), while SHAP values below zero indicate the opposite. ^*^At symptomatic carotid bifurcation on CTA at baseline.

In Figures 7, 8, we show the feature importance using SHAP for the *clinical* (Figure 7) and *combination* (Figure 8) experiments for the prediction of good reperfusion using an RFC model. In this case, the most important features are different from each other when comparing both experiments. While the duration from onset to Intravenous Thrombolysis (IVT), the RR systolic, C-reactive protein (CRP) level, and age seem to be the most important for the *clinical* experiment, these features are all replaced by many radiomics features from multiple brain regions in the *combination* experiment. In addition, this difference can also explain the slightly increased performance of the *combination* experiment when compared with the *clinical* and *image* ones.

### Deep Learning Approach

In Table 4, we present the results for predicting good functional outcome using the *deep learning* approach. To keep the number of experiments feasible, we present results for the *clinical* and *combination* experiments. All the measures were similar for both the *clinical* and *combination* experiments. In the *combination* experiment, training the ResNet10 models from scratch resulted in a worse performance than when using Transfer Learning from other image datasets. Similar to the radiomics approach, there seems to be no improvement in the performance measures when combining the image learned image features with the clinical data (AUC of 0.77 for both). The difference between the results of *clinical* and the *combination* experiments was not significant, *p* = 0.285 for the ResNet10 trained using Transfer Learning.

**Table 4:** Results of all experiments from the deep learning approaches for predicting the good functional outcome [modified Rankin Scale (mRS) ≤ 2].

**Methods**	**AUC**	**F1-Score**	**Sensitivity**	**Specificity**	**PPV**	**NPV**
**Clinical experiment**						
Feed forward	0.77 (0.76–0.78)	0.66 (0.61–0.71)	0.70 (0.66–0.73)	0.70 (0.67–0.74)	0.63 (0.57–0.70)	0.76 (0.72–0.80)
**Combination experiment**						
ResNet10 from scratch	0.54 (0.45–0.64)	0.29 (0.13–0.70)	0.30 (0.20–0.79)	0.78 (0.39–1.00)	0.58 (0.25–0.91)	0.61 (0.51–0.72)
**Combination experiment**						
ResNet10 transfer learning	0.77 (0.75–0.78)	0.66 (0.62–0.70)	0.70 (0.67–0.73)	0.70 (0.68–0.73)	0.63 (0.57–0.68)	0.76 (0.72–0.80)

*Average over 5-fold cross-validation*.

Finally, we present in Table 5 the results for predicting good reperfusion. All evaluation measures are higher for the combination experiment, and, despite often overlapping *CI*s, the average AUC is 0.08 higher. Again, the Transfer Learning approach for the ResNet10 model yielded better results than training from scratch. The difference between the *clinical* and the *combination* experiments was statistically significant, *p* < 0.005 for the ResNet10 trained using Transfer Learning. We provide results from the Delong test in Table 6, which confirms that the difference between the *clinical* and *combinations* experiments was statistically significant.

**Table 5:** Results of all experiments from the deep learning approach for predicting good reperfusion (post-eTICI ≥ 2b).

**Methods**	**AUC**	**F1-Score**	**Sensitivity**	**Specificity**	**PPV**	**NPV**
**Clinical experiment**						
Feed forward	0.53 (0.50–0.55)	0.57 (0.54–0.61)	0.51 (0.48–0.54)	0.53 (0.52–0.55)	0.65 (0.59–0.71)	0.38 (0.32–0.43)
**Combination experiment**						
ResNet10 from scratch	0.50 (0.50–0.52)	0.13 (0.00–0.56)	0.12 (0.00–0.51)	0.87 (0.46–1.00)	0.15 (0.00–0.62)	0.36 (0.31–0.40)
**Combination experiment**						
ResNet10 transfer learning	0.61 (0.50–0.72)	0.63 (0.54–0.71)	0.57 (0.50–0.64)	0.57 (0.50–0.64)	0.69 (0.60–0.80)	0.43 (0.40–0.45)

*Average over 5-fold cross-validation*.

**Table 6:** Delong's test results for the best performing models from eTICI.

**Approach**	**Experiment 1 + model**	**Experiment 2 + model**	**Outcome label**	***P*-value**
Radiomics	Clinical—RFC	Combination—RFC	eTICI	0.04
Deep Learning	Clinical—Feed Forward	Combination—ResNet10 Transfer Learning	eTICI	>0.01

We present ROC curves for the *clinical and combination* experiments for predicting eTICI in Figure 3C. Moreover, we provide the confusion matrices of the deep learning approach for the *clinical* and *combination* experiments (mRs) in Figure 4B and eTICI in Figure 4D.

## Discussion

Our results suggest that there is a statistically significant improvement in discriminative performance for the prediction of good reperfusion (post-eTICI ≥ 2b) when data driven image features were combined with the clinical data, regardless of the approach (radiomics or deep learning). In contrary, the addition of image features does not improve the prediction of good functional outcome (mRS ≤ 2), regardless of the approach. Despite the lack of improvement in prediction accuracy for good functional outcome, radiomics features were relatively important for the models, when viewing 20 features with the highest feature importance.

In the terms of prediction accuracy, our results are in line with previous works on mRS and reperfusion prediction, where AUCs of ~0.80 and 0.57, respectively, were reported (7, 8). The current study is among the first to assess the combination of clinical and image features using both a radiomics and deep learning approaches for the prediction of good functional outcome and reperfusion. A previous study (38) explored a combination of clinical and image data using deep learning approaches to predict the good functional outcome at baseline, and found a significant improvement in the AUC, despite presenting AUC values lower than the ones reported here and in the literature (8). Despite not finding the same improvements as reported (38), our work included a much larger population (3,279 patients vs. 500, respectively). In addition, we performed extra cross-validation iterations, while (38) reported the results for only 1-fold, which might be due to chance.

### Limitations

Several methodological challenges need to be considered when interpreting the results of this study. First, only a relatively small number of deep learning models were used, and they were all based on the same architecture (ResNet10). Second, while other, deeper architectures are available and could yield better results, training a deeper architecture often requires more data and computing power. Since each validation iteration of our experiments takes ~24 h to compute on a single graphics processing unit (GPU) (and deeper 3D architectures would not fit the GPU memory), optimizing other models was out of the scope of this study. Moreover, given the size of our dataset, one could perform more cross-validation iterations, which would make our results more robust. Third, another limitation is the use of CTA modality, while other modalities could also be of added value, such as non contrast CT (NCCT). We chose to use CTA instead of NCCT because previous deep learning studies (12) already found a significant added value of CTAs for predicting the good outcome, but did not explore a 3D approach for the images or their combination with clinical data. Fourth, the large number of variables included can also be a downside, since some are not readily available at baseline, despite all being possible to compute before treatment (either from the patient history or recent imaging).

### Strengths

The strengths of our study include the large and heterogeneous population of patients with LVO as compared with previous studies that aimed at predicting the good functional outcome and reperfusion (7, 8, 12). A heterogeneous dataset is important since we aimed to develop models on data that are as close to the clinical practice as possible. Besides, we employed two different approaches for combining the data, a *radiomics* approach, offering a more interpretable and visual solution, and a *deep learning* approach, which is a more state-of-the-art solution, increasing our chances of finding significant improvements. Another strength of our study is the use of inner and outer cross-validation for optimizing and testing the models. We opted to use a nested-cross validation strategy due to the high risk of bias that having only one single random test set can cause, which can lead to wrong conclusions. By using multiple random test sets (that are unseen during the whole training process), we reduced the risk of random findings due to lucky dataset splits.

### Clinical Interpretation

This study contributes to the understanding of imaging and clinical features that are associated with good functional outcome and reperfusion. Age, NIHSS at baseline, and pre-stroke mRS were found to be the top most important variables for functional outcome prediction, regardless of the data experiment, and have also been found to be relevant in previous studies (7, 8). In addition, with our imaging approaches we identified many relevant brain regions that have also been reported to be significantly associated to functional outcome (16). Predicting the functional outcome at baseline potentially provides the most benefit in clinical practice for assisting decision-making. However, at baseline many prognostic characteristics about the severity of the patient are unknown, making it a challenging task. Baseline variables that capture the severity of patients with stroke (such as, NIHSS and pre-stroke mRS) seem to provide the most insight for prediction, but high predictive values that have been previously reported in the literature (8) can only be achieved when post-treatment variables, such as reperfusion, are available. A perfect prediction of reperfusion could assist the interventionist in their decision to initiate or continue treatment. Moreover, such accurate prediction could assist the prediction of salvaged tissue, and motivate treatment beyond standard inclusion criteria or terminate futile procedures. The prediction of good perfusion for specific treatment devices may support the choice of which device to use during the intervention. The identified variables may furthermore support the understanding of treatments and the improvement of treatment strategies in the near future. Finally, one could consider the prediction of other EVT related outcome, such as treatment complications, that could be of great assistance during EVT.

Regarding feature importance for good reperfusion prediction, despite the poor predictive accuracy, various variables were identified to be relevant. In previous studies the predictors of successful reperfusion have been addressed and resulted in low prognostic value. In these studies, selected device, collaterals, time from onset to IVT, age, ASPECTS, NIHSS, among others, have already been reported to be predictive of reperfusion (39). Nevertheless, despite the risk of chance findings among the variables in Figures 7, 8, most of the variables deemed relevant have either already been reported in the literature, such as duration onset to IVT (40), age (41), occlusion location (42) (M1 occlusions are easier to treat and M2 occlusions increase the risk of symptomatic intracranial hemorrhage), high blood pressure being associated with poor reperfusion (43), among others. Moreover, the relationship between variables should also be taken into account when assessing feature importance, since known predictors, such as time from onset to treatment might have a direct interaction with transfer from another hospital, influencing their impact on the model. Since the main focus of our research was to assess the added value of combining baseline imaging features with the clinical ones, we did not explore the combination with post-treatment variables. Combining baseline images features with post-treatment variables would probably significantly reduce the added value of the (pre-treatment) image features, since the predictive value of post-treatment variables (such as, reperfusion during EVT, duration of EVT procedure, NIHSS at 24–48 h, among others) has already been shown in the literature (8). Nevertheless, such approach could be considered in future models. For future research, one should consider computing more complex radiomics, such as the gray level co-occurrence matrix or shape based features, since these have already been shown to be significantly associated to the outcome of patient with stroke (44). Regarding deep learning, deeper ResNet networks could be considered, provided that enough data are available to train such models, and a Transfer Learning approach should often be explored, since this can greatly surpass models trained from scratch as shown in this study.

## Conclusion

We found a significant improvement in the prediction of good reperfusion when combining image to clinical features. Regarding functional outcome, the addition of image features had no impact on the prediction accuracy. Nevertheless, the prediction accuracy of our models for reperfusion prediction is still rather limited to be considered in clinical practice. The visualization of prediction feature importance showed both known and novel clinical and imaging features with predictive value.

## Data Availability Statement

The data analyzed in this study is subject to the following licenses/restrictions: due to the sensitive nature of the data, the datasets are not publicly available. Requests to access these datasets should be directed to mrclean@erasmusmc.nl.

## Ethics Statement

The studies involving human participants were reviewed and approved by Erasmus Medical Center Rotterdam - MEC-2014–235. The patients/participants provided their written informed consent to participate in this study.

## Author Contributions

LR: lead author, study design, analysis and interpretation, and critical revision manuscript for important intellectual content. HO and AH: study design, analysis and interpretation, and critical revision of manuscript for important intellectual content. AL, YR, WZ, MWa, ME, AZ, GS, and CM: data acquisition and critical revision of manuscript for important intellectual content. AZ, GS, SO, and HM: supervisors of lead author, study design, and critical revision of manuscript for important intellectual content.

## Funding

The MR CLEAN Registry was funded and carried out by the Erasmus University Medical Centre, Amsterdam University Medical, and Maastricht University Medical Centre. The Registry was additionally funded by the Applied Scientific Institute for Neuromodulation (TWIN). ITEA3—Medolution: Project number 14003.

## Conflict of Interest

Erasmus MC received funds from AL. Amsterdam UMC received funds from Stryker for consultations by CM and YR. MUMC received funds from Stryker and Codman for consultations by WZ. CM reports grants from the TWIN Foundation, the CVON/Dutch Heart Foundation, and the European Commission. HM is cofounder and shareholder of Nico.lab. YR and CM own stock in Nico.lab. The remaining authors declare that the research was conducted in the absence of any commercial or financial relationships that could be construed as a potential conflict of interest.

## Publisher's Note

All claims expressed in this article are solely those of the authors and do not necessarily represent those of their affiliated organizations, or those of the publisher, the editors and the reviewers. Any product that may be evaluated in this article, or claim that may be made by its manufacturer, is not guaranteed or endorsed by the publisher.
