# Supplementary material for: Combination of Radiological and Clinical Baseline Data for Outcome Prediction of Patients With an Acute Ischemic Stroke
Source: Front Neurol. 2022 Apr 1;13:809343. doi: 10.3389/fneur.2022.809343 (PMC9010547; doi:10.3389/fneur.2022.809343)
Supplement: Supplementary file 1 [file Table_1.DOCX]

Supplementary Material

**Supplemental Table I.** List of radiomics features computed per atlas region in the radiomics approach. Adapted from the PyRadiomics documentation (17). ROI: Region of Interest.

| Feature Name | Explanation |
| --- | --- |
| 10^th^ Percentile | The 10^th^ percentile of the ROI |
| 90^th^ Percentile | The 90^th^ percentile of the ROI |
| Energy | Energy is a measure of the magnitude of voxel values in an image. A larger values implies a greater sum of the squares of these values. |
| Entropy | Entropy specifies the uncertainty/randomness in the image values. It measures the average amount of information required to encode the image values. |
| Interquartile Range | The 75^th^ minus the 25^th^ percentiles of the image array, respectively. |
| Kurtosis | It is a measure related to the peak of the distribution of values in the image ROI. A higher kurtosis implies that the mass of the distribution is concentrated towards the tail(s) rather than towards the mean. A lower kurtosis implies the reverse: that the mass of the distribution is concentrated towards a spike near the Mean value. |
| Maximum | Maximum gray level intensity within the ROI |
| Mean | Mean gray level intensity within the ROI |
| Mean Absolute Deviation | The mean distance of all intensity values from the Mean Value of the image array). |
| Median | Median gray level intensity within the ROI. |
| Minimum | Minimum gray level intensity within the ROI |
| Range | The range of gray values in the ROI given by: maximum – minimum. |
| Robust Mean Absolute Deviation | Mean distance of all intensity values from the Mean Value calculated on the subset of image array with gray levels in between, or equal to the 10th and 90th percentile |
| Root Mean Squared | Square-root of the mean of all the squared intensity values. It is another measure of the magnitude of the image values |
| Skewness | Measures the asymmetry of the distribution of values about the Mean value. Depending on where the tail is elongated and the mass of the distribution is concentrated, this value can be positive or negative. |
| Total Energy | Total Energy is the value of Energy feature scaled by the volume of the voxel in cubic mm. |
| Uniformity | Measure of the sum of the squares of each intensity value. This is a measure of the homogeneity of the image array, where a greater uniformity implies a greater homogeneity or a smaller range of discrete intensity value |
| Variance | Variance is the mean of the squared distances of each intensity value from the Mean value. This is a measure of the spread of the distribution about the mean. |

**Supplemental Table II.** Hyper-parameters used for optimizing the Machine Learning models using grid-search.

| Classifier | Parameter Name | Parameter Value |
| --- | --- | --- |
| RFC | Number of Trees | [100,200,400,600,800,1000,1200,1400] |
|  | Max features for split | auto, sqrt and log2 |
|  | Max depth of trees | [10,20,30,40, 50, 60, 70, 80, 90, 100, None] |
|  | Quality of split | Gini or Entropy |
|  | Minimum number of samples required to split an internal node | [2,4,6,8] |
|  | Minimum number of samples required to be at a leaf node | [2,4,6,8,10] |
| SVM | Kernel type | Linear, Radial basis function, Polynomial |
|  | Penalty parameter C | [0.001, 0.01, 0.1, 1, 10, 100] |
|  | Kernel coefficient γ (gamma) | [1, 0.1, 0.01, 0.001, 0.0001] |
|  | Degree of the Polynomial kernel | [1,2,3,4,5,6] |
| LR | Regularization | [0.001, 0.01**,** 0.1, 1, 10, 100] |
|  | Optimization algorithm | [newton-cg, lbfgs, liblinear, sag, saga] |
| NN | Hidden Layer sizes | [90,180,90], [90,120,90], [90,90], [90,180], [90], [180] |
|  | Activation | ReLU, logistic |
|  | Regularization parameter | [0.1, 0.01, 0.001, 0.0001] |
|  | Batch size | [32, 64, 128] |
|  | Learning rate | [0.01, 0.001, 0.005] |
|  | Optimization algorithm | Adam |
| XGB | Learning rate | [0.1, 0.01**,** 0.001, 0.005] |
|  | Minimum sum of instance weight (hessian) needed in a child | [1**,** 5, 10] |
|  | Minimum loss reduction required to make a further partition on a leaf node of the tree | [0, 0.5, 1, 1.5, 2, 5] |
|  | Subsample ratio of the training instances | [0.7, 0.8, 0.9, 1.0] |
|  | Parameters for subsampling the columns | [0.3,0.4,0.5,0.6,0.7,0.8] |
|  | Maximum depth of a tree | [3, 5, 7, 9, 10] |

**Supplemental Table III.** Result of the forth experiment (no image score) for the radiomics approach for predicting good functional outcome (mRS<=2). All image-related scores were removed from the clinical data. The average of 5 cross-validation iterations is presented. RFC, random forest classifier; SVM, support vector machine; LR, logistic regression; XGB, gradient boosting; NN, neural networks. AUC, area under the curve; NPV, negative predictive value; PPV, positive predictive value.

| Methods | AUC | F1-Score | Sensitivity | Specificity | PPV | NPV |
| --- | --- | --- | --- | --- | --- | --- |
| Clinical (no image scores) | | | | | | |
| RFC | 0.80  (0.78-0.82) | 0.68  (0.65- .72) | 0.72  (0.68-0.76) | 0.73  (0.71-0.75) | 0.65  (0.61-0.70) | 0.78  (0.76-0.81) |
| SVM | 0.80  (0.78-0.82) | 0.70  (0.66-0.73) | 0.79  (0.73-0.84) | 0.66  (0.65-0.68) | 0.63  (0.58-0.67) | 0.82  (0.78-0.85) |
| LR | 0.80  (0.78-0.81) | 0.69  (0.66-0.73) | 0.77  (0.73-0.81) | 0.68  (0.67-0.69) | 0.63  (0.59-0.67) | 0.81  (0.77-0.84) |
| XGB | 0.79  (0.77-0.81) | 0.68  (0.65-0.72) | 0.75  (0.70-0.80) | 0.69  (0.67-0.70) | 0.63  (0.60-0.66) | 0.79  (0.76-0.83) |
| NN | 0.80  (0.78-0.81) | 0.68  (0.65-0.71) | 0.72  (0.68-0.76) | 0.72  (0.67-0.76) | 0.64  (0.59-0.70) | 0.78  (0.75-0.81) |
| Combination (no image scores) | | | | | | |
| RFC | 0.79  (0.78-0.81) | 0.67  (0.64-0.70) | 0.68  (0.65-0.72) | 0.75  (0.72-0.78) | 0.66  (0.62-0.70) | 0.77  (0.74-0.79) |
| SVM | 0.79  (0.78-0.80) | 0.69  (0.67-0.72) | 0.77  (0.74-0.80) | 0.68  (0.66-0.69) | 0.63  (0.60-0.66) | 0.81  (0.78-0.83) |
| LR | 0.79  (0.78-0.80) | 0.68  (0.65-0.71) | 0.75  (0.70-0.81) | 0.68  (0.66-0.70) | 0.63  (0.59-0.66) | 0.79  (0.76-0.83) |
| XGB | 0.79  (0.77-0.81) | 0.69  (0.66-0.72) | 0.76  (0.74-0.79) | 0.67  (0.65-0.70) | 0.62  (0.58-0.67) | 0.80  (0.78-0.82) |
| NN | 0.72  (0.69-0.75) | 0.60  (0.55-0.64) | 0.60  (0.54-0.66) | 0.71  (0.69-0.73) | 0.59  (0.55-0.64) | 0.71  (0.68-0.75) |

**Supplemental Table IV.** Results of the forth experiment (no image score) for the radiomics approach for predicting good reperfusion (post-eTICI ≥ 2b). All image-related scores were removed from the clinical data. The average of 5 cross-validation iterations is presented. RFC, random forest classifier; SVM, support vector machine; LR, logistic regression; XGB, gradient boosting; NN, neural networks. AUC, area under the curve; NPV, negative predictive value; PPV, positive predictive value.

| Methods | AUC | F1-Score | Sensitivity | Specificity | PPV | NPV |
| --- | --- | --- | --- | --- | --- | --- |
| Clinical (no image scores) | | | | | | |
| RFC | 0.51  (0.48-0.54) | 0.70  (0.67-0.73) | 0.77  (0.72-0.82) | 0.25  (0.21-0.30) | 0.64  (0.59-0.68) | 0.39  (0.33-0.46) |
| SVM | 0.53  (0.51-0.55) | 0.75  (0.66-0.83) | 0.92  (0.72-1.13) | 0.09  (0.16-0.33) | 0.64  (0.60-0.67) | 0.43  (0.19-0.61) |
| LR | 0.53  (0.52-0.55) | 0.63  (0.58-0.68) | 0.61  (0.56-0.66) | 0.42  (0.38-0.46) | 0.64  (0.60-0.69) | 0.39  (0.36-0.42) |
| XGB | 0.51  (0.50-0.52) | 0.64  (0.58-0.71) | 0.65  (0.54-0.76) | 0.39  (0.31-0.47) | 0.64  (0.60-0.69) | 0.40  (0.36-0.44) |
| NN | 0.52  (0.49-0.55) | 0.76  (0.71-0.80) | 0.94  (0.83-1.05) | 0.07  (0.06-0.20) | 0.63  (0.59-0.67) | 0.43  (0.23-0.64) |
| Combination (no image scores) | | | | | | |
| RFC | 0.57  (0.54-0.60) | 0.74  (0.71-0.76) | 0.87  (0.84-0.89) | 0.17  (0.13-0.21) | 0.64  (0.60-0.68) | 0.42  (0.33-0.52) |
| SVM | 0.56  (0.53-0.60) | 0.65  (0.54-0.75) | 0.66  (0.42-0.90) | 0.41  (0.12-0.70) | 0.66  (0.62-0.70) | 0.42  (0.35-0.49) |
| LR | 0.56  (0.53-0.59) | 0.61  (0.59-0.64) | 0.57  (0.55-0.60) | 0.49  (0.44-0.54) | 0.66  (0.62-0.70) | 0.40  (0.35-0.46) |
| XGB | 0.56  (0.54-0.59) | 0.60  (0.57-0.64) | 0.55  (0.52-0.59) | 0.53  (0.48-0.57) | 0.67  (0.63-0.70) | 0.41  (0.36-0.46) |
| NN | 0.52  (0.50-0.55) | 0.66  (0.64-0.69) | 0.70  (0.60-0.79) | 0.34  (0.22-0.46) | 0.64  (0.59-0.69) | 0.39  (0.33-0.45) |
